# C-reactive Protein is an Independent Predictor of Difficult Emergency Cholecystectomy

**DOI:** 10.7759/cureus.4573

**Published:** 2019-04-30

**Authors:** Gordon C Gregory, Matta Kuzman, Jayaram Sivaraj, Alex P Navarro, Iain C Cameron, Glen Irving, Dhanwant Gomez

**Affiliations:** 1 Hepato-Pancreato-Biliary Surgery, Nottingham University Hospitals, Nottingham, GBR

**Keywords:** cholecystectomy, cholelithiasis, choledocholithiasis, cholecystitis, pancreatitis

## Abstract

Purpose

The objective of this study was to identify variables that predict a difficult laparoscopic cholecystectomy performed in an emergency setting. The secondary aim was to devise a pathway for patients admitted acutely that required a cholecystectomy.

Methods

Patients admitted to the Emergency General Surgery Department at Nottingham, the United Kingdom that had an emergency cholecystectomy performed during the one-year period from May 2016 to June 2017 were identified. Collected data included patient demographics, clinical presentation, biochemical analysis, radiological findings, subsequent interventions, surgical data, and clinical outcome. A difficult cholecystectomy was defined as operative time >60 minutes, conversion to an open procedure, or sub-total cholecystectomy performed.

Results

A total of 149 patients were included. Cholecystitis was the most common diagnosis (*n *= 86, 57.7%), followed by acute pancreatitis (*n *= 36, 24.1%). Fifty-five (36.9%) patients had an elevated C-reactive protein (CRP) >100 mg/dL. One hundred and twenty-one (81.2%) patients who had an emergency cholecystectomy were defined as “difficult”. The overall morbidity rate was 15.4% (*n *= 23), and there was no post-operative in-hospital mortality. Univariate analysis showed that age >60 years (*p *= 0.012), underlying diagnosis (*p *= 0.010), presence of heart rate >90 (*p *= 0.027), and an elevated pre-surgery CRP >100 (*p *< 0.001) was associated with a difficult emergency cholecystectomy. Multi-variate analysis demonstrated that an elevated pre-surgery CRP >100 was an independent predictor of a difficult emergency cholecystectomy (*p *= 0.041).

Conclusions

An elevated pre-operative CRP is an independent predictor of a technically more difficult cholecystectomy in the emergency setting.

## Introduction

Current published data that include meta-analyses of randomised controlled trials are in agreement that emergency cholecystectomy in acute cholecystitis is more cost-effective than delayed cholecystectomy without increased risk of complications or conversion to open cholecystectomy [1-3]. In addition, studies have also shown that emergency cholecystectomy is associated with similar post-operative morbidity and shorter length of hospital stay compared to elective cholecystectomy following discharge from their index admission [4]. Nevertheless, there is still a wide variation in the management of patients presenting with acute gallbladder pathology. The reason for this is likely to be multi-factorial and usually both patient- and/or hospital-related factors are often contributing factors for these differences. In a recent UK study that included almost 9,000 patients who underwent a cholecystectomy, only 16% of cases were performed as an emergency and almost half (47%) of the patient cohort had their cholecystectomy performed in an elective setting [5]. 

It was previously thought that early or emergency cholecystectomy was associated with increased morbidity and mortality rates and increased conversion rates to an open procedure [6]. However, recent meta-analyses have concluded that there are no significant differences in clinical outcome following cholecystectomy performed as an emergency compared to an elective setting [1,3]. However, there are demographic and clinical factors that can increase the difficulty in performing an emergency cholecystectomy and increase the risk of specific complications. Recently, Stromberg and Sandblom demonstrated that patient co-morbidity and poly-pharmacy increased the risk of bleeding following cholecystectomy [7]. Other investigators have also shown that obesity, previous upper abdominal surgeries, comorbid diseases such as diabetes mellitus, acute cholecystitis, raised white cell counts, increased gallbladder wall thickness, presence of peri-cholecystitic collection, contracted gallbladder and adhesions at Calot’s triangle was associated with a difficult cholecystectomy [8]. 

C-reactive protein (CRP) is an acute-phase reactant protein secreted by the liver in response to pro-inflammatory cytokines in the presence of inflammation, infection, trauma and underlying malignancy [9-10]. The CRP circulating concentration is a good indicator of the severity of inflammation [10]. Mok et al. observed that patients with gangrenous cholecystitis had a significantly higher CRP, with a CRP level >200 mg/dL having a 50% positive and 100% negative predictive value for gangrenous cholecystitis [11]. Other investigators have also shown that besides an absolute value (>190 mg/dL), interval change in CRP (increase >90mg/dL) at 48 hours from admission predicts the severity of pancreatitis [12].

The aim of the present study was to identify clinical variables that predicted a difficult laparoscopic cholecystectomy performed in an emergency setting in patients during their acute admission. The secondary aim was to devise a pathway for patients admitted acutely that required a cholecystectomy, either as an emergency or in an elective setting.

## Materials and methods

Patients admitted to the Emergency General Surgery Department at the Nottingham University Hospitals NHS Trust, Nottingham, the United Kingdom, who had an emergency cholecystectomy performed during the one-year period from May 2016 to June 2017, were identified from the hospital’s operating theatre (Bluespier) database. Exclusion criteria included paediatric patients (less than 16 years old). This project has been registered and approved by the institutional board for Clinical Audit in Surgery (project number 18-321c).

During this study period, a dedicated specialized hepato-pancreato-biliary (HPB) consultant-led emergency surgery assessment for all patients with HPB pathology was introduced in the Trust [13]. Collated data included patient demographics, clinical presentation, biochemical analysis, radiological findings, subsequent interventions, surgical data and clinical outcome. 

The lowest systolic blood pressure and highest heart rate of the patient within 24 hours of surgery were recorded. The laboratory measurement of serum samples was recorded for liver function tests (LFTs) including alanine aminotransferase (ALT, 0-35 U/L), aspartate aminotransferase (AST, 0-30 U/L), alkaline phosphatase (ALP, 40-130 U/L) and bilirubin (0-21 μmol/L). Serum lipase (0-78 U/L) samples were recorded as the first measurement made during their hospital admission. Urea (2.9-7.5 mmol/L), electrolytes (sodium: 134-145 mmol/L and potassium: 3.5-5.3 mmol/L), and estimated glomerular filtration rate (GFR, normal >90) were also determined. In addition, the pre-operative (day before surgery) CRP (0-10 mg/l) and white cell count (WCC, 4-11x109/l) were recorded. 

Radiological investigations were reviewed and reported by consultant radiologists. Radiological dilatation of the common bile duct (CBD) was defined as greater than 7 mm on ultrasound (US), computed tomography (CT) and magnetic resonance cholangiopancreatography (MRCP) [14]. 

Cardiorespiratory co-morbidities were classified between one and four using the P-POSSUM criteria. Frailty was also scored as per the Charlson age-comorbidity index.

The four primary categories of diagnosis were biliary colic, cholecystitis, cholangitis and pancreatitis. Pancreatitis was categorized biochemically if there was an elevation in serum lipase (4x the normal limit) or radiologically demonstrated on CT. Biliary colic was differentiated from cholecystitis if the CRP and WCC were within the normal laboratory range and the gallbladder wall on US was thin-walled with no evidence of surrounding inflammation. Cholangitis was diagnosed in the presence of sepsis with an elevation in CRP and WCC levels as well as a raised serum bilirubin. 

A cholecystectomy was defined as difficult if the operative time was sixty minutes or more, required an open procedure or conversion to an open procedure, or resulted in a sub-total cholecystectomy due to inability to dissect Calot’s triangle safely. Other investigators have used similar definitions [15]. All cholecystectomies were either performed by the consultant HPB surgeon on-call or supervising a senior surgical trainee performing the procedure. Clinical outcomes included the length of hospital stay, morbidity and mortality. Specific complications from cholecystectomy including bile leak, re-operation and infection were recorded. Other outcomes include operating time, retained CBD stones, conversion to an open procedure and re-admissions. 

The project was completed in accordance with the guidelines set by the Strengthening of Reporting of Observational Studies in Epidemiology (STROBE) statement for observational studies. Data was collected and analysed in the relevant categories.

The abstract of this article has been presented in poster format (Poster: Gregory G: C-reactive Protein as an Independent Predictor of Difficult Emergency Cholecystectomy: AUGIS Scientific Meeting 2018: Edinburgh 19th-21st September 2018).

Statistical analysis

Categorical data weres presented as frequency and proportions (%) and were analysed using Pearson’s chi-squared test or Fisher’s exact test, with significance taken as *p *< 0.05. Median and range were used to describe continuous data. Statistical analysis was performed to assess for a significant difference in demographics, diagnosis, comorbidities, laboratory tests and outcome between patients that underwent a standard cholecystectomy and a difficult cholecystectomy. All statistical analyses were performed using SPSS for Windows™ version 16.0 (SPSS Inc, Chicago, Ill, USA), and statistical significance was taken at the 5% level.

## Results

**Figure 1:**
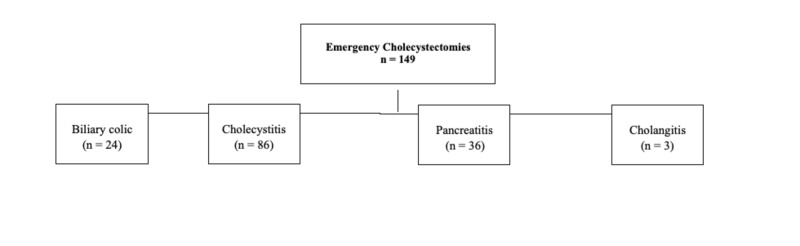
Algorithm of the outcome of patients in this study

During the study period, 149 emergency cholecystectomies were performed. One hundred and six patients (71.1%) were female and the median age of patients was 48 (range: 16-85) years. There were 70 (47.0%) patients who had a frailty score of more than 0. Cholecystitis was the most common diagnosis (*n *= 86, 57.7%), followed by acute pancreatitis (*n *= 36, 24.1%; Figure 1).

Fifty-five (36.9%) patients had an elevated CRP >100mg/dL. US showed the presence of a thick-walled gallbladder in 75 (50.3%) patients, while CBD dilatation was observed in 27 (18.1%) patients. Fifteen (10.1%) patients underwent an endoscopic retrograde cholangio-pancreatography (ERCP) for stone extraction prior to their cholecystectomy (Table 1). 

**Table 1:** Statistical analysis of variables with respect to patients undergoing emergency cholecystectomy

Demographic, clinical and pathological factors		Standard cholecystectomy	Difficult cholecystectomy	Uni-variate analysis	Multi-variate analysis	Risk ratio (Confidence Interval)
		(n = 28)	(n = 121)			
Age	> 60 years	3 (10.7%)	43 (35.5%)	0.012	0.238	2.112 (0.611-7.302)
Gender	Female	23 (82.1%)	83 (68.6%)	0.173		
Co-morbidities	Cardiac	3 (10.7%)	24 (19.8%)	0.413		
	Respiratory	1 (3.6%)	13 (10.7%)	0.470		
Frailty score	>0	10 (35.7%)	60 (49.6%)	0.161		
Diagnosis	Biliary Colic	9 (32.1%)	15 (12.4%)	0.010	0.150	1.889 (0.794-4.493)
	Cholecystitis	11 (39.3%)	75 (62.0%)			
	Pancreatitis	8 (28.6%)	28 (23.1%)			
	Cholangitis	0 (0%)	3 (2.5%)			
Ultra Sound Findings	Thick walled Gall Bladder	11 (39.3%)	64 (52.9%)	0.167		
	CBD dilated	5 (17.9%)	22 (18.1%)	1.000		
ERCP	Pre Surgery	0 (0%)	15 (12.4%)	0.075		
Pre-surgery physiology	HR >90	2 (7.1%)	32 (26.4%)	0.027	0.345	2.055 (0.461-9.150)
	Systolic BP <110 / >130	9 (32.1%)	48 (39.7%)	0.523		
Admission to cholecystectomy	>3 days	11 (39.3%)	60 (49.6%)	0.325		
Elevated pre-operative blood tests	Bilirubin	5 (17.9%)	32 (26.4%)	0.468		
	Alkaline phosphatase	12 (42.9%)	74 (61.2%)	0.089		
	Alanine transaminase	13 (46.4%)	69 (57.0%)	0.289		
	White cell count	8 (28.6%)	48 (39.7%)	0.387		
	CRP >100	2 (7.1%)	53 (43.8%)	<0.001	0.041	4.919 (1.066-22.704)
Post-operative	Complications	3 (10.7%)	20 (16.5%)	0.440		
	Re-admissions	4 (14.3%)	12 (9.9%)	0.500		

The median final pre-operative heart rate recorded was 80 (52-124) bpm, and the median lowest systolic blood pressure recorded pre-operatively was 123 (92-108). One hundred and twenty-one (81.2%) patients who had an emergency cholecystectomy were defined as “difficult”. Eleven (7.4%) patients had a conversion to an open procedure. A sub-total cholecystectomy was performed in five (3.4%) patients. The overall median operating time was 92 (28-226) minutes. There were 121 (81.2%) patients whose cholecystectomy was more than one hour of the operative time. Thirty-five (23.5%) patients had on-table cholangiograms and five (3.4%) patients had CBD exploration, of which two cases were performed laparoscopically.

Twelve (8.1%) patients required high dependency admission post-cholecystectomy, of which 11 patients had a difficult cholecystectomy. The overall morbidity rate was 15.4% (*n *= 23), and there was no post-operative in-hospital mortality (Table 2). Repeat surgery was required in four (2.7%) patients for bleeding (*n *= 1), bile leak (*n *= 1), and collections (*n *= 2). Re-admission occurred in 16 (10.7%) patients. 

**Table 2:** Complications and re-admission of patients following emergency cholecystectomy in this study

Complications and Re-admissions	Total (n)
Complications	23 (15.4%)
Intra-abdominal collection	6
Retained Stone	6
Bleeding	3
Wound Infection	3
Bile Leak	1
Pancreatitis	1
Delerium	1
Pneumonia	1
Ileus	1
Re-admissions	16 (10.7%)
Post-operative pain	6
Intra-abdominal collection	4
Wound infection	2
Retained common bile duct stones	4

Statistical analysis

Univariate analysis showed that age >60 years (*p *= 0.012), underlying diagnosis (*p *= 0.010), presence of heart rate >90 (*p *= 0.027) and an elevated pre-surgery CRP >100 (*p *< 0.001) were associated with a difficult emergency cholecystectomy. Multivariate analysis demonstrated that an elevated pre-surgery CRP>100 was an independent predictor of a difficult emergency cholecystectomy (*p *= 0.041) in this study.

## Discussion

At present, the current published evidence would support emergency cholecystectomy for gallstone-related complications [1]. Studies have shown that emergency cholecystectomy has similar conversion rates to open cholecystectomy and potentially associated with less time off work compared to an elective cholecystectomy due to patients having their definitive management earlier [16-17]. Nevertheless, the delivery of this service would be dependent on the institution. In general, specialist HPB centres are associated with higher volumes of emergency cholecystectomy. This may be due to the assumption that an emergency laparoscopic cholecystectomy is presumed to be technically more difficult compared to a standard elective cholecystectomy. 

Variables associated with a difficult cholecystectomy

 In the present study, the majority of the patient cohort was classified as a difficult emergency cholecystectomy. On univariate analysis, this study showed that patients aged 60 years or more were more likely to have a difficult emergency cholecystectomy. The groups of Constantini and Lee identified advanced age (>60 years) as a significant risk factor for intraoperative difficulty and conversion to an open procedure in their studies [18-19]. Another significant variable in univariate analysis in this study was the underlying diagnosis. A difficult cholecystectomy was more likely to be associated with the diagnosis of cholecystitis, pancreatitis or cholangitis compared to biliary colic. Other investigators have also reported similar findings [19-21]. Recently, Tan et al. concluded that delayed cholecystectomy was associated with lower conversion to open rates and shorter length of hospital stay in patients with acute cholecystitis presenting beyond seven days of symptoms compared to emergency cholecystectomy [22]. Although the presence of pre-operative tachycardia was associated with a difficult cholecystectomy on univariate analysis, this could be related to the underlying diagnosis.

 An elevated pre-surgery CRP>100mg/dL was an independent predictor of a difficult emergency cholecystectomy in the present study. Portinari and co-workers observed that a high CRP was associated with severe acute cholecystitis, and these patients may benefit from an emergency cholecystectomy [23]. Teckchandani and co-investigators observed that a raised CRP on admission in cases of acute cholecystitis was associated with a higher emergency laparoscopic converted to an open cholecystectomy procedure [24]. Recently, Mok et al. concluded that the CRP level during acute admission was an important predictor of a difficult cholecystectomy and also conversion to an open procedure [25]. The authors showed that more than two-thirds of patients who had a CRP>220mg/dL during their index admission were converted to an open procedure irrespective whether the cholecystectomy was performed as an emergency or in the elective setting.

It is important to be able to predict a difficult cholecystectomy and determine a patient cohort that is likely to benefit from having a laparoscopic cholecystectomy in the emergency setting and the experience required to perform it. Recently, the Dutch Pancreatitis Study Group observed that a difficult cholecystectomy after mild gallstone pancreatitis could be predicted if the patient was of male gender, had prior sphincterotomy and had a delayed cholecystectomy [15]. In an analysis of 22,953 patients, Giger et al. observed an increase in morbidity in cases of difficult laparoscopic cholecystectomy, which were associated with prolonged operative time and increased conversion to an open procedure [26]. In contrast, the current study did not observe an increase in morbidity in the difficult cholecystectomy group. Besides surgical factors such as consultant experience, patient selection is crucial in also determining cases that should be done as an emergency. 

Limitations

There are limitations to this study. This was a retrospective study, with a dedicated specialized HPB on-call system. In addition, the collected data represented a 1-year period of practice in a tertiary centre, and hence the small sample size. Although there were more patients classified as a difficult cholecystectomy, this could be due to the fact that the majority of patients were diagnosed with acute cholecystitis, and had surgery for this indication as well as for pancreatitis and cholangitis compared to biliary colic. Furthermore, a “difficult” cholecystectomy can be considered subjective, and based on the operating surgeon. Hence, in this study, in an attempt to quantify and assess difficulty in performing a cholecystectomy surrogate markers were used: duration of the procedure; conversion to an open procedure; or a sub-total cholecystectomy performed. 

**Figure 2:**
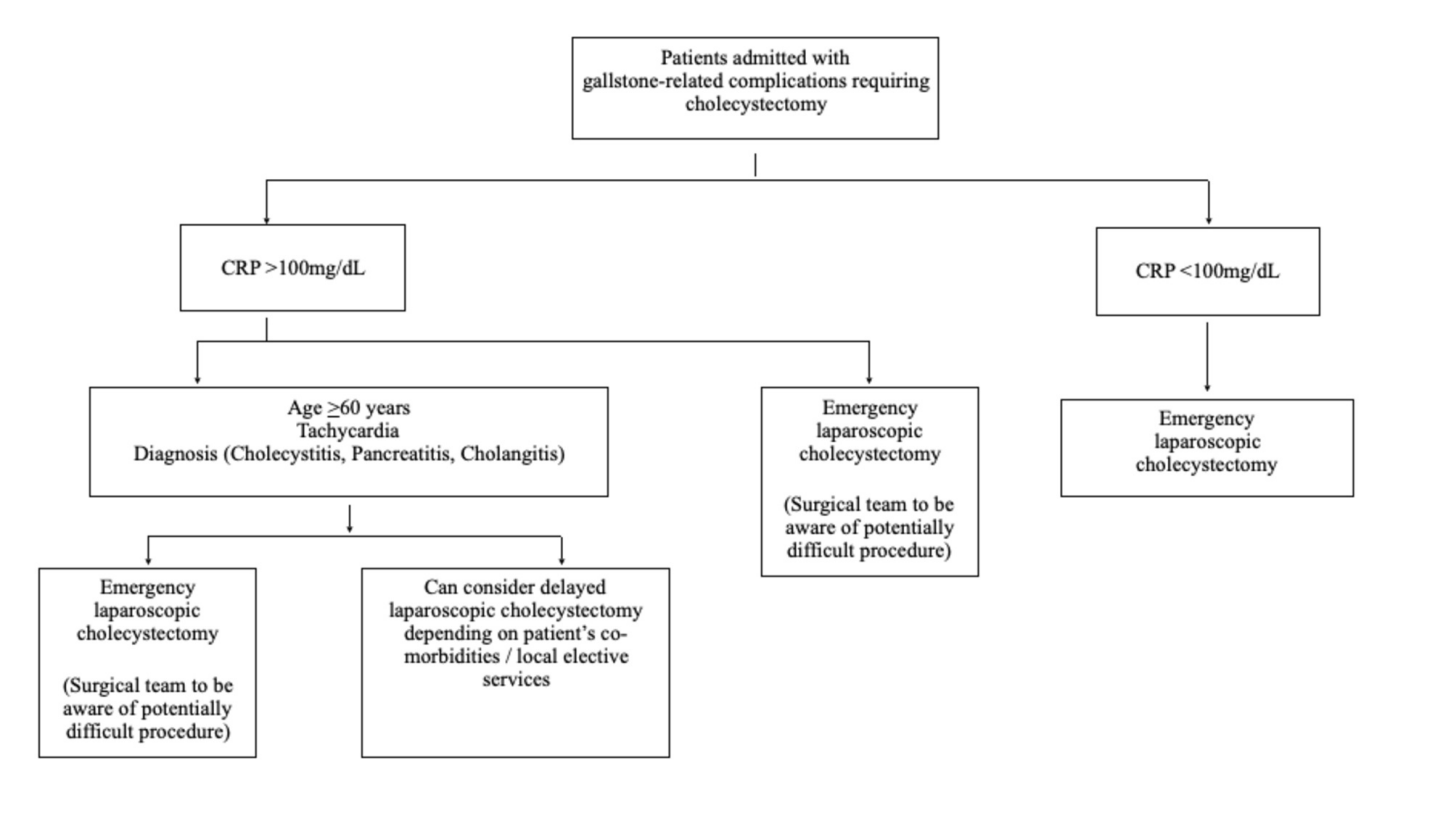
Suggested algorithm for patients presenting as an acute surgical admission with gallstone-related complications that require an emergency cholecystectomy

Due to the retrospective nature of this study, and the focus on patients undergoing an emergency cholecystectomy, variables measured included laboratory results, physiology status, radiological parameters and intra-operative findings. All cholecystectomies performed had the involvement of the HPB consultant on-call, during the decision-making process as well as the surgical procedure. The above reasons could account for the variation that may be observed in other centres. Surgical decision-making process depending on consultant sub-specialty, the complexity of other surgical admissions and emergency operating volume are potential contributing factors that may influence the delivery of emergency cholecystectomy in a particular institution [27-28]. A proposed algorithm for emergency laparoscopic cholecystectomy was constructed based upon results from the present study (Figure 2).

## Conclusions

An elevated pre-operative CRP is an independent predictor of a technically more difficult cholecystectomy in the emergency setting. Patient selection is crucial to determine which sub-group of patients will benefit from having an emergency cholecystectomy.
